# A Neuro-Phenomenological Perspective on the Autism Phenotype

**DOI:** 10.3390/brainsci11070914

**Published:** 2021-07-10

**Authors:** Antonio Narzisi, Rosy Muccio

**Affiliations:** IRCCS Stella Maris Foundation, 56018 Pisa, Italy; rosymuccio@yahoo.com

**Keywords:** autism, spirituality, health, sensoriality, cognitive priors, mindfulness

## Abstract

In the current paper, we present a view of autism spectrum disorder (ASD) which avoids the typical relational issues, instead drawing on philosophy, in particular Husserlian phenomenology. We begin by following the recent etiological perspectives that suggest a natural predisposition of a part of individuals with ASD towards hypersensitivity and the reduced influence of cognitive priors (i.e., event schemas). Following this perspective, these two characteristics should be considered as a sort of phenomenological a priori that, importantly, could predispose people with ASD towards a spiritual experience, not intended in its religious meaning, but as an attribute of consciousness that consists of being aware of and attentive to what is occurring in the present moment. Potential clinical implications are discussed.

## 1. Introduction

The autism spectrum disorder (ASD) is a neurodevelopmental disorder characterized by a symptomatic dyad represented by an impairment of social interaction and the presence of restricted and stereotyped interests and behaviors [1]. Recent epidemiological data attest to the presence of this disorder in 1 out of 54 subjects [2,3], effectively freeing ASD from being a rare disease to ascribe it to a condition with high social impact. The etiology of ASD remains unknown to date, although scientific data confirm that the cause is to be found in a complex multifactorial relationship [4].

In the face of the recognized symptom dyad that has described ASD for over 50 years [5], at least two other interrelated peculiarities delineate the autistic phenotype. The first is hypersensitivity [6] and the second is the reduced adherence to cognitive priors [7]. Both of these characteristics, as we shall see, are those that can allow us to reflect on the relationship between ASD and spirituality [8,9].

## 2. Hypersensitivity

Baron-Cohen [10] traced the recognized attention to detail exhibited by people with ASD to sensory hypersensitivity. To explain sensory hypersensitivity, Mottron and Burack [11] developed the model of enhanced perceptual functioning in persons with ASD to emphasize their superior bottom-up perceptual processing. Studies using questionnaires such as the Sensory Profile have revealed sensory abnormalities in over 90% of people with ASD [6,12,13]. Regarding vision, Bertone and Faubert [13] showed that individuals with ASD are more accurate on visual discrimination tasks. Regarding the auditory modality, higher discrimination and processing ability has been found in individuals with ASD compared to control groups consisting of individuals without autism [14,15,16,17]. In the area of tactile modality, Blakemore et al. [18] showed hypersensitivity to vibrotactile stimulation at a frequency as low as 200 Hz. Two studies have investigated olfaction in ASD, and unlike the research on the other senses that consistently find hypersensitivity, both of these studies reported deficits in odor identification [19,20]. However, viewing the two studies more closely, both required participants to accurately identify the odor from a choice of responses, and therefore this task involved both executive function and memory. Sensory hypersensitivity could result from a difference in processing at various levels that could include hypersensitivity of sensory receptors, dysregulation of excitatory and inhibitory neurotransmitters, or increased speed of neural processing. In this line of research, Belmonte et al. [21] have hypothesized that the peculiar sensory hypersensitivity found in people with ASD seems to be due to a neural hyperconnectivity at short distance as opposed to a hypoconnectivity at long distance.

Regarding this last point, Kessler, Seymour, and Rippon [22] showed that the findings from subsequent research have been inconsistent, with a lack of agreement about the nature of any high-frequency disturbances in ASD brains. Based on the application of new techniques using more sophisticated measures of brain synchronization and direction of information flow, and invoking the coupling between high- and low-frequency bands, they propose a framework which could reconcile apparently conflicting findings in this area and would be consistent both with emerging neurocognitive models of ASD and with the heterogeneity of the condition.

## 3. Reduced Adherence to Cognitive Priors

Cognitive priors are a set of mental schemas that daily orient people who do not have ASD towards an unconscious inference about the structure of the world that, on the one hand, is consistent with the retinal image transmitted to the brain and, on the other hand, is conditioned by memories (cognitive priors/mental schemas). In people with ASD, the reduced adherence to cognitive priors is the feature that makes them perceive the world in a “real” way without cognitive models of the form/gestalt of the world. Studies of perception have highlighted, through the central coherence theory [23], that in people without ASD, knowledge of the world is organized around perceptual gestalts, or rather our brain tends to process an overall meaning rather than perceive the details that contribute to create the whole [24]. Frith and Happé’s [23] central coherence deficit hypothesis was the first to suggest that nonsocial symptomatology in ASD could be explained by a perceptual style that privileges details and individual parts. People with ASD have a natural tendency to perceive details rather than the figure as a whole [25].

In particular, this hypothesis suggests that a specific perceptual-cognitive style, loosely described as a limited ability to understand context or to “see the big picture”, underlies the central disturbance in ASD.

For example, on the *hidden figure test*, people with ASD showed abilities above average in detecting elements that are well nested in a context. The reason a person without ASD struggles with the task is that the context overrides the elements that make up the whole. In the perception of the person without ASD, context prevails because cognitive priors (mental schemas of the shape of the world) significantly (and evolutionarily) orient knowledge of reality. Following this line of research, in 2019, we tested how children with ASD use contextual priors [26]. We tested this hypothesis within the action domain in children with and without ASD using a behavioral paradigm comprising a familiarization and a testing phase. During familiarization, children observed videos depicting a child model performing actions in diverse contexts. Crucially, within this phase, we implicitly biased action-context associations in terms of their probability of co-occurrence. During testing, children observed the same videos but drastically shortened (i.e., reduced amount of kinematics information) and were asked to infer the action unfolding. Since during the testing phase, movement kinematics became ambiguous, we expected children’s responses to be biased to contextual priors, thus compensating for perceptual uncertainty. While this probabilistic effect was present in controls, no such modulation was observed in children with ASD, overall suggesting an impairment in using contextual priors when predicting other people’s actions in uncertain environments.

The perceptual characteristics of people with ASD have also been studied through optical illusions. Since many illusions are due to the influence of global context on the components, a detail-oriented and less context-influenced perception helps to avoid being misled by the illusion [24]. Perceptual studies continue to provide evidence for a perceptual style that is detail-based rather than ensemble-based and suggest that in people with ASD, superior perceptual ability in detail discrimination may interfere with the development of other abilities and be influenced by the reduced adherence to cognitive priors.

## 4. Between Spiritual Dimension and Phenomenological Experience

Marked hypersensitivity and independence from pre-existing mental schemas are interconnected and mutually influential characteristics. In people with ASD, the reduction of mental schemas means that there is greater exposure to sensory influences, which in turn are less conditioned by these schemas. This peculiar neurosensory dynamics of some autistic phenotypes could represent, as phenomenology argues, a scientific penetration of life that could open the way to the spiritual dimension. In this work, the spiritual dimension is not intended in its religious meaning but as an attribute of consciousness that consists of being aware of and attentive to what is occurring in the present moment [27].

However, while for the phenomenologist, the spiritual experience cannot even be thought of without the philosopher’s disposition to challenge the obvious and, at the same time, without the methodological rigor of the scientist [28], in the person with ASD, the spiritual experience in the phenomenological sense seems to constitute a biological predisposition, an a priori, rather than a conscious challenge to the obvious. In other words, while for the phenomenologist, the *epoché*, defined by Husserl [28] as a total suspension of presuppositions and of the being-already-given-of-the-world (i.e., cognitive priors) and as a fundamental step for reaching the transcendental level [29], is neither an intellectual artifice nor a mystical enlightenment but constitutes a methodical and laborious operation, for the person with ASD, the *epochè,* so understood, could be considered as a natural predisposition. Following this hypothesis, some people with ASD could already be predisposed not to have strong and universal constraints in perception, and therefore, they appear less influenced by the being-already-given-of-the-world (i.e., cognitive priors). In this sense, the person with ASD does not need to neutralize the complex of mental habits (i.e., cognitive priors) that in subjects without ASD could represent an interruption of contact towards the spiritual experience in a phenomenological sense.

From a practical point of view, the *epochè* is considered the basis of meditation practices that appear to be linked to a state of well-being, such as mindfulness [30].

In fact, as reported by Crescentini et al. [27], an important quality of mindful awareness is the promotion of non-judgmental awareness of the self in which activated self-feelings, thoughts, and sensations are not attempted to be changed by the perceiver but are instead observed and accepted (i.e., epochè). Mindfulness awareness, and its cultivation through meditation, would operate through erosion of habitual patterns of responding (i.e., cognitive priors) and of the use of evaluative language and thinking; this would promote self-insights and a greater acceptance of one’s internal states, including intuitive feelings, potentially leading to the transformation of implicit self-attitudes and perhaps to a better tuning of one’s implicit and explicit self-representations.

Mindfulness-based programs for children with high-functioning ASD are showing that children, including adolescents, improve their emotional and behavioral functioning, increasing mindful awareness on all occasions. There are good prospects, to be validated by research, for considering mindfulness a practice that can help people with ASD (probably high-functioning) to organize, enhance, and make functional their natural predisposition to transcendence.

## 5. Conclusions

In this work, the view of ASD has not focused exclusively on relational issues [31,32,33]. Rather, the complexity and the extreme variability of ASD have been viewed in light of new etiological perspectives that have highlighted a neurophysiological predisposition for hypersensitivity and reduced influence of cognitive priors/schemas.

In the future, it would be interesting to investigate, through evidence-based research, if what we have defined as the phenomenological a priori (i.e., hypersensitivity and reduced cognitive priors) could play a role during self-awareness trainings (i.e., mindfulness) in terms of activation of specific brain networks. The literature, conducted on subjects without ASD, indicated that spiritual experiences linked to meditation are underpinned by a large brain network [34,35].

This line of research, if adequately developed, would constitute a new and stimulating field of study and would be in line with what is proposed by the World Health Organization about the relationship between the spiritual dimension of health and clinical/therapeutic outcome [36,37].

## Data Availability

There are no data in this paper.
